# Experiences of operational managers regarding record keeping by new professional nurses in public hospitals in the North West province, South Africa

**DOI:** 10.4102/hsag.v28i0.2257

**Published:** 2023-08-25

**Authors:** Naomi L. Nkoane

**Affiliations:** 1 Department of Health Studies, College of Human Science, University of South Africa, Pretoria, South Africa

**Keywords:** documentation, intentional recording, mindful recording, operational managers, patient records, recording

## Abstract

**Background:**

Documentation can be written or computerised and is used to communicate healthcare and treatment among healthcare professionals. Documentation is the tool that records and measures the healthcare provided to patients, and it must be accurate, complete and timely.

**Aim:**

This study aims to explore and describe the experiences of the operational managers regarding record keeping by new nurses in selected public hospitals in South Africa.

**Setting:**

The study was conducted in selected public hospitals in the North West province.

**Method:**

This study used a qualitative, explorative and descriptive approach with a purposive sampling method. A total of 35 operational managers participated in the process of data collection.

**Results:**

The following themes emerged from this study: gaps in record keeping, the impact of inaccurate documentation and the need for improvement in record keeping.

**Conclusion:**

The study has shown the need to bring technological innovation to strengthen the effective improvement of digitalisation in nursing record keeping in the facilities furthermore, nurses should be supported through programmes on intentional and mindful record keeping curbing the incidences of inaccuracy and incompleteness.

**Contribution:**

This study’s findings confirmed that new nurses were not consistent with accurate documentation of patient records, and this needs further strengthening in public hospitals to have an impact on the health and safety of the patient.

## Introduction

Documentation of records is a critical component for the functioning of nursing teams and cannot be detached from excellence of patient care (Firouzeh et al. 2016). Patient health records are significant for the provision of healthcare services (Mutshatshi et al. 2018). Documents should be able to present the patient’s health assessment, present any treatment rendered and guide health professionals on how to deal with planned care.

Failure to provide evidence of recorded care delivered to patients denotes that patient care was either not given or was inadequate. Documentation of health records is regarded as instrumental in assisting with the timeous recognition of any changes in the patient’s condition; it enables communication and continuity of care (Shihundla, Lebese & Maputle 2016). Record keeping leads to shared communication among the health professionals and, ultimately, prompts intervention (Andualem et al. 2019).

Mutshatshi et al. (2018) emphasised that comprehensive, timeously and accurate record keeping serves as the best nursing practice. The keeping of good health records is critical in the formation of the long-term account of the patient’s illness and comprehensively enhances assessment of their health needs (Abdelrahman & Abdelmageed 2014). Improved practices of nursing documentation can reduce morbidity and mortality of patients in healthcare facilities. Additionally, it is also important that recording becomes an individual professionals’ best interest in the clinical practice and that time should be allocated in the working day activities for the maintenance of documentation (Beach & Oates 2014).

Several studies have shown that recording of patients’ information increases nurses’ workload, and as a result, they fail to comprehensively capture information (Al-Kandari & Thomas 2009; Ayele et al. 2021; Shihundla et al. 2016). Brima et al. (2021) determined that several challenges inhibited quality documentation of patients’ health conditions; this included insufficient, unclear and missing information in the files. Dimond (2013) commented on the lack of clarity and no recording of action taken in response to the identified problems as common mistakes occurring in the documentation.

Bizimana and Bimerew (2021) reported in their Burundian study that nurses showed adequate knowledge and positive attitudes towards the recording of patients’ health information. Andualem et al. (2019) believed that despite the knowledge and positive attitudes of Ethiopian nurses towards nursing documentation, record keeping continues to be a global challenge. Barriers were also noted as impacting negatively on the provision of quality nursing care. Furthermore, Ayele et al. (2021) echoed the importance of improving the working environment through increasing the nursing staff to manage the workload in healthcare facilities so that records can be effectively completed.

Literature on comparison of paper-based and electronic health records have both shown notable and equitable challenges (Akhu-Zaheya, Al-Maaitah & Bany Hani 2017). Convergently, the overall content recording of nursing care plans, whether paper-based or electronic system, yielded the same results (Wang, Yu & Hailey 2015). The study by Hariyati et al. (2020) revealed that electronic nursing documentation was effective in the improvement of patients’ care. Interestingly, an Iranian study found that the vocal-electronic systems improved the quality of document recording in nursing (Firouzeh et al. 2016).

The South African healthcare facilities could benefit from the use of these types of systems where a nurse speaks to an electronic device which then captures all the care carried out for the patient, thus improving the quality of nursing records. The study by Mirzaeian, Mobasheri and Khaledifar (2013) concluded that developing nations can benefit from the mobile technologies to promote effective and efficient healthcare provision. Nurses should be trained in becoming tech-savvy to navigate the systems and portals that can make their lives easier in comparison to the manual system of documenting patients’ records. Verma and Gupta (2016) declared that the nurses needed training on electronic health records in their units.

Technological applications designed specifically for the documenting of health records could make a big difference in nurses’ day-to-day tasks. As a result, challenges such as lost and damaged files would be avoided, resulting in improved communication among health teams, which in turn will prompt better patient interventions, and patient care and safety will be achieved.

The nurse managers’ efforts to improve documentation of health records in the facilities have not gone unnoticed and have now become a persistent worldwide challenge (Okaisu et al. 2014). However, nurse–patient ratio also contributes to the widening gap of insufficient documentation resulting in majority of patients’ records remaining either incomplete or undocumented (Kebede, Endris & Zegeye 2017).

Nurses are using technology when rendering patient care daily, for example, the monitoring of blood pressure. This, however, becomes an incomplete process when after rendering electronical care, nurses manually record actions taken using pen and paper. According to Carayon and Hoonakker (2019), it is significant to evaluate how best human factors and organisational aspects can be embraced to improve the integration of the workflow in the units and the positive impact on clientele health safety and medical outcomes. Therefore, electronic data storage can be well managed and kept permanently using available systems like that of Electronic Health Record (EHR) systems (Ross, Wei & Ohno-Machado 2014).

Substantial scholarly work has been done on record keeping in healthcare facilities and that impacts negatively on the delivery of quality healthcare services. Unfortunately, the classical literature and contextual gaps have been noted. The classical literature gaps are on lack of intentional and mindful record keeping, which has not been studied. As such the concept of mindfulness in record keeping can ring opportunities to improve on the quality record keeping. The contextual gap is shown by evidence of less work conducted on record keeping by new professional nurses in North West province (NWP). Thus, record keeping remains an unanswered and unresolved problem, and further research opportunities are critical on the studied phenomenon.

## Problem statement

Despite the significance of medical records within the healthcare system, documentation of patients’ health matters is still a global challenge (Wu et al. 2019). According to Prideaux (2011), record keeping has generally failed to maintain the recommended standards, which has, in turn, contributed to poor quality nursing care. Record keeping of nursing care provided to patients ensures quality and continuity of care through communication among healthcare professionals (Mathioudakis, Rousalova & Gagnat 2016). Kasaye et al (2022) reported that more than average of medical services was unrecorded, whereas completion of recording on the client’s health condition is necessary for the delivery of quality healthcare.

Operational managers in the selected public hospitals in the NWP were concerned about poor record keeping by the new professional nurses in their units during routine internal audits. Based on the background above, this study seeks to explore and describe the experiences of the operational managers regarding record keeping by new professional nurses in public hospitals in the NWP, South Africa.

## Research aim

The aim of this study was to explore and describe the experiences of the operational managers regarding record keeping by new professional nurses in selected public hospitals in the NWP, South Africa. Following the introduction, this study presented the research methods that were undertaken and discussed them in detail, followed by the presentation of the study results with no articulation of the literature review. Then, the study presents the discussion of the results and how they link to the literature, and finally, it presents a conclusion drawn from the results indicating possible areas for further research. Thus, this study reports on the challenges of record keeping and recommends effective solutions for remediation. Furthermore, a qualitative approach was considered as appropriate for answering the research question posed about record keeping – ‘What are the challenges regarding patients’ record keeping the health units?’

## Methods

### Research design

The study adopted a qualitative, explorative and descriptive research design. A constructivist paradigm was followed to establish a meaning of the topic under study from the participants’ viewpoint (Creswell & Creswell 2018). This approach enabled the researcher to better understand the phenomenon under study from the perspective of the 35 randomly selected operational managers and how they experienced record keeping in their units in the public hospitals of NWP in South Africa.

### Research setting

The study was conducted on operational managers in selected public hospitals in the NWP. The hospitals that granted permission to conduct the study were found within two districts of the NWP. The researcher carried out the study in a naturalistic setting as the interest focus was on the operational managers’ experiences of the phenomenon (Polit & Beck 2021). The role of these managers is to provide administrative oversight in the units to deliver quality healthcare.

### Population and sampling

Polit and Beck (2021) defined population as all the individuals or objects with common defining characteristics. The study population consisted of all operational managers employed permanently in selected public hospitals in the NWP. A convenient purposive sampling was used to choose a certain representation of the study sample, with a central focus on four focus group discussions (FGDs), each consisting of 8 and 12 participants (Gray, Grove & Sutherland 2017). The researcher wanted to understand the reasons why keeping of records by nursing personnel has become a chronic challenge in various health facilities. Focus group members verbally agreed and signed that they will not share the research information with anyone. The researcher ensured that the hospital names remain anonymous and instead the numbers were used, for example, participant 2, 7, 9 …, to protect the identity of the participants. The data collected during study were not shared with any unauthorised personnel for confidentiality of the participants’ information.

The inclusion criteria comprised of operational managers who were on duty on the day and time of data collection. Furthermore, operational managers who are permanently appointed in the posts at the hospitals and who had been in the post for 2 years and more were included. Exclusion criteria included those who had been employed for less than 2 years or who were in acting positions.

### Data collection

Data collection was conducted in a naturalistic setting, public hospitals, over a period of 6 weeks.

The researcher began with laying the ground rules for each session of FGD for smooth data collection. The purpose of data collection was explained, and the participants consented to the process by signing consent forms to affirm that they were participating in the study out of their own free will. They also consented to the focus group interviews being audio recorded. The seating arrangement of the participants was in a circular pattern to allow for face-to-face engagement between the researcher and the participants. Data collection was guided by the use of interview guide; this consisted of a major question followed by probing and follow-up questions. The aim of the FGDs was to obtain rich data from the participants in their own setting. All the FGDs lasted on average, for an hour, and fieldnotes were also captured. Data collection was conducted until saturation was reached at the fourth FGD (Polit & Beck 2021).

### Data analysis

Data analysis ran concurrently with data collection using the six steps of the Clarke and Braun (2013) framework. The researcher transcribed the recordings verbatim from audio to text. Data analysis commenced with: (1) familiarisation of data, with the researcher re-reading through the transcripts and notes; (2) generation of initial codes to collate data; (3) themes were tagged to pick up patterns; (4) themes were reviewed by searching for relationships between repeated ideas in the documents; (5) themes were defined and named; and (6) finally, the report was produced. The researcher established 3 themes, 6 categories and 14 sub-categories from the data analysis.

### Measures to ensure trustworthiness

The trustworthiness of the study was anchored on the framework of the Lincoln and Guba (1985) as cited by Polit and Beck (2021). The following trustworthiness criteria were followed: credibility, dependability, confirmability and transferability. To ensure the credibility of the study, the researcher extended reasonable time in the field with the nurse managers during the FGDs to gain an in-depth understanding of their challenges. Existing truths of the nurse managers and the evidence of the audio recordings are available on request. Moreover, fieldnotes were captured to ensure credibility of this study. Confirmability in this study was grounded using a co-coder to confirm that the codes were aligned to the themes, audio recordings and the field notes.

Additionally, the researcher maintained dependability through a documented research audit trail that was kept for the benefit of other scholars interested in equivalent studies on patients’ records. Lastly, the study further ensured dependability through the bracketing of the researcher’s own preconceived ideas and beliefs to reduce bias (Polit & Beck 2021). The author during data collection ensured that she remained aware of personal biases and own values as part of knowledge and avoided introducing own preferences through keeping notes in a journal format to enhance the quality of qualitative data collection. Thus, the researcher maintained that she approached data in its purest form without influence of personal values and conflict of interest (Polit & Beck 2021). To satisfy transferability, the study used multiple settings of four district hospitals, and thus, the findings may be transferred to public regional and provincial tertiary hospitals in the NWP.

### Ethical considerations

Ethical clearance was obtained from the University of South Africa (90388526_CREC_CHS_2021) prior to the commencement of data collection, followed by obtaining permission from the North West Department of Health and the District Managers. Meetings were arranged with the hospitals to meet with the participants to brief them about the information of the study and its methodologies on the ethical issues related to this study. Informed consents were signed by the participants before any actual data collection commenced; however, no names were attached to maintain confidentiality. Participants were informed before the data collection about their right to self-determination and that they are free to terminate their participation from the data collection without prejudice (Gray et al. 2017). Finally, the operational managers were not coerced into participating in the data collection process (Polit & Beck 2021). The sessions were recorded based on the permission of the participants with one of the main reasons being to assist the researcher with transcription for data analysis.

## Results

Table 1 details the demographics of the participants who were willing to share their stories with the researchers.

**TABLE 1:** Demographics of participants.

Hospitals number	Classification of hospital	Gender		Age		Ethnicity		Managerial experience	
		F	M	> 40 years	< 40 years	Black people	Other	> 5 years	< 5 years
Hospital 1	District	10	3	2	11	9	4	1	12
Hospital 2	District	9	0	0	9	9	0	0	9
Hospital 3	District	6	1	6	1	7	0	0	7
Hospital 4	District	6	0	0	6	3	3	0	6

**Total**	**-**	**31**	**4**	**8**	**27**	**28**	**7**	**1**	**34**

F, Female; M, Male.

A total of 35 nurse managers from four selected district hospitals participated in the FGDs. Female participants dominated the sessions in all hospitals, with 31 females out of 35 participants confirming that nursing is mainly a feminine profession. There were only four male participants with just one being below the age of 40 years. Ethnic grouping of the participants was diversified, with black females in higher numbers, followed by white females, then mixed race females, black males and one mixed race male. The managerial experience of the participants varied from 4 to 10 years.

Three main themes emerged from the data collected from the focus groups: namely, gaps in document keeping, impact of inaccurate documentation, and improvement in record keeping. Themes, categories and sub-categories presented in Table 2 are discussed in the next section.

**TABLE 2:** Themes, categories and sub-categories established in relation to documentation by nursing personnel.

Theme	Category	Sub-category
1. Gaps in record keeping	1.1 Individual factors	Knowledge of documentation Attitudinal behaviour Professionalism
	1.2 Incomplete records	Principles of record keeping Unfinished task recording Erroneous patients’ records
2. Impact of inaccurate documentation	2.1 Provision health care	Shortage of staff Increased workload Policies and protocols
	2.2 Nursing practice	Legal and ethical implications Poor nursing care
3. Improvement in record keeping	3.1 Significance of documentation	Guidance Mentoring
	3.2 In-service trainings	Competence in record keeping

### Theme 1: Gaps on record keeping

The above-mentioned theme is associated with the actual care given to the patient and that record keeping is recommended as best practice in healthcare facilities. Failure to record the healthcare of patients amounts to providing poor quality healthcare. Most of the nurse managers described the documentation and missing information on patients’ health conditions, patients’ treatment and care as the challenge they are faced with daily in their units:

‘There are lot of gaps. I don’t know if you will agree with me, report-writing is a challenge. Everything that you do, you must write it down. There is a saying that if you did not write it, you did not do it. Even if you did a hundred things and you did not write any, it is not done. Seemingly report-writing, documentation, it’s lacking everywhere.’ (FGD 3, female, Participant 5)

#### Individual factors

These factors refer to the behaviours observed by the operational managers from the nurses working in their units:

‘These nurses should show that he or she is somebody who is well- trained and knowledgeable.’ (FGD 3, female, Participant 6)

Participants determined that nurses are well trained. Consequently, the participants acknowledge that nurses should have knowledge of documentation and execute their duties regarding record keeping.

**Knowledge of documentation:** The nurse managers alluded to the fact that nurses are not well grounded in the importance of documentation knowledge and stated the following:

‘Record-keeping is very important in rendering health care. Nurses should know better about keeping complete records because we are the one spending most time with the patients, but we are lacking with record keeping.’ (FGD 4, female, Participant 4)

‘Professional nurses need to have this interest in their work. Passion in the workplace is very important. They should have knowledge of the importance of record. They communicate with everybody freely through record keeping and should feel confident about what they know. New nurses should not rely on their seniors all the time, they were trained and have knowledge. Now, new professional nurses must know the importance of record keeping, they are also supervisors of the lower categories in the unit.’ (FGD 3 female, Participant 5)

Most of the participants concluded that professional nurses were not mindful of recording important information about the patient’s healthcare in addition, they are not passionate about their work. This fed into the following category in which most of the participants reported a lack of enthusiasm towards record keeping.

**Attitudinal behaviour:** Documentation in the nursing profession is a fundamental and integral component of the field. Healthcare professionals have a responsibility to adopt a positive attitude and show passion towards records to ensure that they are kept according to the standard required. Participants alluded to the existence of negative attitudinal behaviour among nurses:

‘Now they are having attitude. The attitude towards recording exists because some will respond with “I do not know” or are dismissive. They are careless with the records and most of the time there are missing pages. They do not want to be corrected. If you correct them, they become hostile.’ (FGD 3, female, Participant 5)

Participants perceived negative attitude towards documentation by new professional nurses, which led to a feeling that recording in general was a burden to them because they felt that it took too much time and attention away from patient care.

#### Incomplete records

Most of the participants reported that records were incomplete and as such undermined any intended communication among the health professionals which can cause harm to the patients. One of the participants stated that:

‘In my [*maternity*] ward, I also did documentary analysis. Our partographs are always incomplete. Hence there are so many litigations, we are losing women and their babies in the wards. This is a critical key area that we need to put more focus on.’ (FGD 4, Female, Participant 3)

Many of the participants indicated that incomplete records remain a persistent challenge in the units, and basically, it undermines the credibility of new nurses regarding their understanding of principles of recording.

**Principles of record keeping:** Most of the participants described record keeping of nurses as failing to reflect the principles of record keeping:

‘Record-keeping is very important, they should make sure that it reflects the principles of recording, and it should be legible, with the date and the time included just to cover themselves.’ (FGD 2, Female, Participant 3)

The study showed that lack of knowledge about the basic principles of record keeping, consequently, impacted the quality of recording because nurses were unable to complete records of the tasks they have performed for the patients.

**Unfinished task recording:** Furthermore, the study participants found that the recording of tasks was often left unfinished and such actions were picked up by managers when they conducted spot audits in their units. Some of the participants also reported an element of dishonesty from the nurses about their tasks. The following accounts are verbatim quotes from the responses of the participants:

‘I will go through the medication charts, just to see if documentation was done correctly, for example, injections? I will also look at the doctor’s orders if they were carried out? If they are documented? You will find that such task is unfinished, meaning that the task is 50 percent recorded. Basically, it is not done.’ (FGD 4, Female, Participant 5)

‘And I will check for the signatures to confirm completeness. Signature represents that the person takes the responsibility of what has been done on the patients but sometimes the records are not signed.’ (Hosp 3 P4, FGD 4, Female, Participant 7)

‘Unfinished tasks is common and what is essence of half tasks.’ (FGD 3, Male, Participant 4)

‘They are also not honest with execution of tasks. When I check the file, nothing, not even phenytoin was not even prescribed, but she said the patient was loaded … that thing of not being honest also is a challenge. Then asked her what did you do? She said the patient was given medication but there is no signature on the treatment chart. The patient was not even given a thing … it was not even prescribed. There was only Valium that was prescribed, when necessary, and it was never given since the patient was admitted, that is being dishonest.’ (FGD 3, Female, Participant 7)

The study showed that patients’ documents were incomplete and displayed unfinished tasks, because nurses failed to attach their signatures as affirmation of taking responsibility and accountability of the patient care and interventions rendered. Not only unfinished tasks but also errors in records were noticed by the participants.

**Erroneous patients’ records:** Most of the participants reported frequent errors in the patients’ plan of care. Participants noted the errors as very serious and that they are related to inability to cope with their work in the units. Furthermore, lack of coping may result in additional complicating ill-health of the patients:

‘If they document, it is either erroneous, or it does not appear on the patient file.’ (FGD 4, female, Participant 7)

‘Maybe they are not coping, because the records have mistaken, which can lead to giving wrong treatment thus worsening illnesses.’ (FGD 1, female, Participant 4)

The first theme revealed gaps identified with record keeping of health and nursing care in the units of the public hospitals. Thus, disregarding proper record keeping seems to be a health hazard.

### Theme 2: Impact of inaccurate documentation

This theme refers to the clear, accurate and accessible documentation as an element of safe, quality and evidence-based nursing practice (American Nurses Association [ANA] 2010). Documentation of nurses’ work is critical for effective communication with each other as well as with other disciplines (ANA 2010). Participants stated that inaccurate record keeping has a negative bearing on nursing care provided to the patients. One of the participants recorded that:

‘I won’t just sit there and say I’m a manager when it comes the life of a human being, and for this patient I’ll stand up and I’ll make sure and check. Even if not doing their job, I will just check, be in there to see if they are doing their job correctly, monitoring them, and check if the patient now is being transferred to the higher level, is everything done and recorded correctly to avoid possible litigations as they are very costly for the Department.’ (FGD 1, Female, Participant 5)

Failing to keep quality records of patient care may be the cause of inadequate provision of care, as discussed below. Additionally, inaccurate documentation may result in medical litigations that deplete the departmental budgets.

#### Provision of healthcare

This category was discussed in relation to the failure in keeping quality records, resulting in the poor provision of nursing care. All the participants acknowledged the factors contributing to quality record-keeping. Participants reported that because of interrelated factors such as shortage of personnel, nurses tend to cut short their activities and recording thereof. One of the participants shared that:

‘Due to the shortage of staff and sometimes there are two or three nurses on duty, we do not have the time and staff to execute all activities. Then nurses sort to the shortcuts because they must do hundred things and you have maybe twenty minutes for the hundred things, and then it tends to … the recording shortfalls comes in and it affects the nursing care.’ (FGD 1, Female, Participant 5)

The study revealed that there were attributable factors to nurses’ work that impacted on recording shortfalls, thus affecting the delivery of nursing care to the patients. Participants indicated that staff shortage was one the factors that had a direct impact on the recording of nursing care in the public hospitals.

**Shortage of staff:** Participants have indicated that the actual number of nurses was far less than the proposed nurse-patient ratio. Most of the participants reported that this may influence the quality of documentation. Nevertheless, all the participants echoed the importance of record keeping despite shortage of nurses. This is what one of the participants had to say:

‘There is shortage of staff, but you cannot say I failed to record data on the patients’ file because there was a shortage of staff. What did you do as a professional?’ (FGD 1, Female, Participant 7)

Additionally, participants highlighted work overload as a secondary factor to the shortage of staff. They also acknowledged and noted that both the shortage of staff and increased workload lead to documentation errors.

**Increased workload:** This sub-category speaks about inadequate staffing, which, in turn, led to the inability to smoothly give nursing care in the units, and eventually, health recording was neglected. Participants have acknowledged the fact that their units are always full, most likely nurses are failing to record because of workload. Some participants shared the following:

‘The patients that we have in the units are many and that leads to increased workload.’ (FGD 2, Female, Participant 7)

‘We have not managed to keep up with increasing demands of the health care services and our profession suffers mostly because patients are coming in numbers but with few nurses available, thus increased burden on the record keeping.’ (FGD 4, Female, Participant 4)

Participants revealed that the increased workload contributed to nurses finding themselves not keeping up with or ‘cutting corners’ of the existing policies and protocols within the nursing profession. Mistakes can often be made when taking shortcuts, and eventually, the nursing is compromised.

**Policies and protocols:** As most participants realised that recording was a challenge, they echoed the need to go back to basics and use the nursing care plans as done previously. Participants raised the need to give attention to the simplest and most important matters even though they have ignored the basic principles of record keeping. Some narratives from the participants in the FGDs have been captured:

‘I think we should go back to basics – use the nursing care plans as evidence-based nursing.’ (FGD 2, Female, Participant 1)

‘They should know why recording is important to us nursing personnel and other health professional, recording protocols are very clear but they are not implemented.’ (FGD 3, Female, Participant 5)

Thus, participants alluded that adherence to the fundamental principles of recording would improve nurses’ record keeping as well as improve nursing practice and patient satisfaction with the nursing care given.

#### Nursing practice

The participants spoke about nursing practice as being the field of indirect and direct patient care in units that promoted health and reduced health problems and assisted in maintaining optimal health through the good practice of documented patient health records. However, participants also pointed out that the legal and ethical aspects of such non-completion.

**Legal and ethical aspects:** Most of the participants reported that undermining accuracy and completeness of patients’ records may pose a patient safety risk, resulting in disciplinary and legal action as this is considered negligence:

‘Failure to record is the main driver for disciplinary and litigations we see most of the time.’ (FGD 4, Male, Participant 6)

‘Discipline is lacking nowadays, time has changed., however we need to go back to basics. Nursing ethics need to be strengthened, maybe things may improve.’ (FGD 1, Female, Participant 11)

‘Teaching nursing etiquette in the units is neglected and we must strengthen that gap. We should not be weary of teaching and supporting them [*nurses*].’ (FGD 1, Female, Participant 1)

Seemingly, most participants alluded that discipline is lacking among new professional nurses. All participants reported that they should rise to the call of action to teach professional nurses the importance of recording and constantly support them to reduce legal and ethical effects relating to poor record keeping. In addition, they stated that it is the nurses’ legal and professional duty to keep a comprehensive data record of their patients.

**Poor nursing care:** The participants described sub-par nursing care as being malpractice when referring to the service that the nurses provide. The outcomes of care provided resulting from sub-standard documentation, can lead to inadequate monitoring, improper diagnosis and eventually unnecessary care. Some of the excerpts from the FGDs included:

‘Report-writing and documentation, it’s lacking everywhere. That may lead to compromised nursing care. So, if we can enhance that through continuing with teaching them the right things.’ (FGD 3, Female, Participant 7)

The study participants concluded that inadequate recording results in poor nursing care, and hence, the necessity of an improvement in record keeping is significant. This is addressed within the last theme below.

### Theme 3: Improvement in record keeping

This theme reports mainly on strategies to improve the challenges of documentation in the units. Most of the nurse managers emphasised that provision of a knowledge development programmes would empower nurses on the importance of record keeping, and improved record keeping would thus be achieved. Participants declared that tradition of ignoring record keeping is rising, and therefore, constant monitoring and support are necessary. These are some of the statements voiced by the participants:

‘When it comes to our documentation it does appear that there is tradition of overlooking importance of record keeping.’ (FGD 3, Female, Participant 5)

‘I will just check, be in there to see if they are doing their job correctly, monitoring them, and check if the patient is being transferred to the higher level as is supposed to, is everything done correctly? Is documentation part of it? So that it will gives our hospital a good image.’ (FGD 4, Female, Participant 2)

Nurse managers revealed that it is their role to ensure that new professional nurses are supported on the importance of keeping complete and accurate records of the patients.

#### Significance of documentation

This category addresses the ability of nurses to remain educated about the importance of best practice in the documentation of patient health matters in their workplace. The accuracy of the records directly relates to the quality of healthcare treatment, planning and its execution in the units. Participants vowed that they will always explain to the nurses the importance of record keeping. One of the participants extracts:

‘I always explain how important recording is to them, and that whatever you are doing you must record it, because if you don’t record anything that simply means you didn’t do it. And if you are not sure about something, ask first before you give the wrong things.’ (FGD 1, Female, Participant 7)

Participants emphasised the need for continued guidance and mentoring of nurses as vital to empowering them and developing their understanding of the significance of record keeping and, ultimately, instilling in them the need for consistency of accurate records.

**Guidance:** Most of the participants emphasised that there is still a need to guide professional nurses, especially those who are new entrants in the profession. Furthermore, nurse managers challenged each to lead by example, be present among nurses and avoid delegating the tasks always. These are some of their voices:

‘I’m a manager but when it comes the life of a human being, [*patient*] I’ll stand up and make sure that I check if all things are in order. I am not doing their job nor policing but just to provide guidance.’ (FGD 1, Female, Participant 8)

‘It is important that we supervise them.’ (FGD 4, Female, Participant 1)

‘Don’t just delegate and sit there and … delegate something that you also know that you’ve got the knowledge of it. Lead by example.’ (FGD 4, Female, Participant 5)

Participants encouraged each other to continue to take their leadership roles in the guiding and mentoring of nurses in their units and be present during the provision of nursing care and recording thereof, and not to always remain in their offices.

**Mentorship:** Most of the participants indicated that it is imperative to augment mentorship programmes to support their nurses with regard to patient record keeping in the units. The nurse managers reported the following:

‘They need support all the time and our trainings.’ (FGD 2, Female, Participant 9)

‘You are a supervisor that you must always support and educate, also check, and be part of the team- spot teaching.’ (FGD 3, Female, Participant 4)

Nurse managers reflected on the benefits of persistent professional growth and development, especially spot-teaching. They also advocated for trainings to improve job outcomes.

#### In-service trainings

In addition to attending mentorship programmes, nurses should be encouraged to also attend ongoing in-service trainings to improve on the challenges of record keeping they are currently faced with in the units. Some of the study participant voiced that:

‘They must also show passion in learning, in attending training.’ (FGD 3, Female, Participant 7)

‘Allow them to attend all these trainings, the forums, the conferences. These trainings are valuable spacing to allow for knowledge to shared and boost their confidence.’ (FGD 1, Female, Participant 8)

Nurse managers emphasised on training attendance. They were of the opinion that professional nurses will become knowledgeable and confident, if they are well trained.

**Competence in record keeping:** The participants acknowledged that there are system challenges such as understaffing and overworked nurses which compromise competence in record keeping. However, they stressed that nurses should still maintain good record keeping. One of the participants said:

‘… Not exactly but the question is here: how can record keeping competence of the professional nurses be enhanced? As the operational managers, what are we doing to change the situation now? When you find cases where patient has died and when you read the notes there was no resuscitation commenced. The patient’s notes only captured “called a doctor to certify or to see an unresponsive patient. We need to support them to the level of competency in documentation”.’ (FGD 4, Female, Participant 5)

Seemingly, operational managers have reported that record keeping in the units has been a regular challenge because there is consistent evidence of incompetency and non-compliance with the principles of recording in general. The study recommends that nurses should strive to improve record keeping values and practices to attain document quality.

## Discussion

The study findings indicated that the documentation of patients’ records has been insufficient and inadequate in the sampled public district hospitals, as documented by various scholars earlier also (Johnson, Edward & Giandinoto 2018; Olivier & Kyriacos 2011; Tasew, Mariye & Teklay 2019). Although a substantial number of scholars have written on the drivers and barriers of documentation (Mutshatshi et al. 2018; Muyakui, Nuuyoma & Amukugo 2019; Prideaux 2013), this study reports that the nurse managers perceive the challenges of record keeping as ongoing. Documentation in the units should reflect critical thinking of the nurses in the planning of care for patients (Kamil, Rachmah & Wardani 2018).

The participants described individualistic factors associated with the knowledge gap and attitude towards documentation. In this study, the knowledge of nurses regarding documentation seemed unclear, as it is displayed by the attitudes of the nurses when it comes to recording nursing care given to patients. An earlier study by Andualem et al. (2019:9) indicated that nurses with a positive attitude towards documentation are likely to have good documentation practice. This study has shown inaccurate recording and that nurses neglected their recording obligation of patient care which resulted in the omission to identify early signs of deterioration or changes in the health conditions of the patients. Additionally, the study by Vincent et al. (2018) denoted that prompt detection of complications resulted in appropriate patient management and improved the chances of survival.

The study findings articulated challenges relating to the inadequacy of the documentation, and these were mostly associated with training of nurses, similar to the finding supported by Kamil et al. (2018). The study found that nurses do not understand the significance of documentation of patients’ records, as most of the vital data were missed in the nursing care plans. Nurses failed to keep records and tasks were incomplete, and these were attributed to increased workloads (Al-Kandari & Thomas 2009; Kebede et al. 2016). Not only incompleteness of records was observed but also erroneous recording. This non-compliance to the principles of record keeping is compounded by the competing activities associated with the demands of the healthcare system.

Furthermore, supervisors of the units should ensure that documentation is strengthened and assessed continually to improve patient safety and cost-effectiveness (Asmirajanti, Hamid & Hariyati 2019). However, diverging findings by Nakate et al. (2015) have shown that the nurses have a positive attitude towards the documentation of patient care, but limiting system’s pressures were noted.

Quality documentation of nursing care has been reported in the current study and by other several scholars as crucial for the provision of quality care, continuity of care and patient safety. Furthermore, missed nursing care recording is associated with low staffing numbers in the hospitals (Bjerkan, Valderaune & Olsen 2021; Griffiths et al. 2018). Managing clinical workload within nursing units is the responsibility of the nurse managers (Hegney et al. 2018). It is important that nurses become cognisant of the legality and ethical aspects of records in the health field, despite the existing drivers of non-compliance in the units such as increased workload and shortage of nurses.

Finally, issues of policies and protocols were also articulated with the emphasis that nurses should embrace existing guidelines to do right by capturing patient’s histories, thus avoiding any possibility of legal and disciplinary processes among nurses. Failure of nurses to record nursing care rendered to the patients equals sub-standard care and even leads to fatalities. Introduction of technological software applications to support record keeping might improve on the documentation gaps and increase effective recording keeping (Firouzeh et al. 2016).

Therefore, further research focusing on improvement strategies is critical for contextual alleviation of the challenges experienced by the nurse managers in their settings (Bunting & De Klerk 2022). Consistent support and guidance have not been underrated; the current findings are largely reported on continuous professional development to increase awareness of the understanding of existing prescripts and protocols on the documentation of health records (Bjerkan et al. 2021). The good recording of information is necessary for effective nursing practice, which is then dependent upon the quality of the information captured by the nurses (Urquhart et al. 2018). The study further highlighted the competence gap and the need for this to be bridged through in-service trainings to improve patient safety. Similar sentiments were shared by Law, Akroyd and Burke (2010) as well as Rahmah, Hariyati and Sahar (2022).

### Strengths and limitations

The findings were only based on the exploration of the experiences of the operational managers regarding record keeping by new professional nurses in selected public hospitals in the NWP, South Africa. Therefore, the findings of this study cannot be generalised to other nurse managers in other provinces. In addition, maintaining research integrity through application of ethical considerations and trustworthiness enhances the rigour of the study.

### Recommendations

Failure of record keeping implies that the principle of ubuntu and that of record keeping are not taken into consideration during patient care in the healthcare facilities. Therefore, there is a need for formal in-service training for all new professional nurses on the importance of record keeping. There is also a need for innovative way of responding to the problem at hand. For such a time as this of fourth industrial revolution, time is now to explore digitalisation of record keeping through the involvement of the National Department of Health and Department of Technology to collaborate and design technological software specific for record keeping. It is imperative to bring innovative approaches to strengthen and improve digitalisation in nursing record keeping in healthcare facilities. Furthermore, nurses should be supported through programmes to address intentional and mindful record keeping using technology applications to curb the incidences of inaccuracy and incompleteness. Nursing Education Institutions should ensure that intimacy of recording keeping begins during training of the students, so that it is strengthened through teaching and learning. This may assist in bringing solutions to reduce the challenges of record keeping and ultimately improve the quality of healthcare in public hospitals.

## Conclusion

The evidence indicates that nurse managers are experiencing difficulties with the documentation of the patients’ health conditions in their units. Although they acknowledge that system challenges do exist, documentation should be prioritised as it has a direct influence on the outcomes of nursing care provided to patients. Therefore, programmes of support to improve on the challenges of documentation are urgently needed. These support programmes will strengthen the capacity building of the nurses regarding intentional and become mindful of how important record keeping is and improve the quality of healthcare in public hospitals. Finally, the use of technology may assist in improving record keeping in the public hospitals.
